# Complications and Management of the Thoracic Endovascular Aortic Repair

**DOI:** 10.1055/s-0040-1714089

**Published:** 2020-11-05

**Authors:** Sheena W. Chen, Kyongjune B. Lee, Michael A. Napolitano, Alejandro E. Murillo-Berlioz, Anna P. Sattah, Shawn Sarin, Gregory Trachiotis

**Affiliations:** 1 George Washington University Hospital, Washington, District of Columbia; 2 Holy Cross Hospital, Silver Spring, Maryland

**Keywords:** thoracic aorta endovascular repair, endovascular, aortic disease, thoracic aorta, complications, management, stent graft

## Abstract

Endovascular treatment in thoracic aortic diseases has increased in use exponentially since Dake and colleagues first described the use of a home-made transluminal endovascular graft on 13 patients with descending thoracic aortic aneurysm at Stanford University in the early 1990s. Thoracic endovascular aneurysm repair (TEVAR) was initially developed for therapy in patients deemed unfit for open surgery. Innovations in endograft engineering design and popularization of endovascular techniques have transformed TEVAR to the predominant treatment choice in elective thoracic aortic repair. The number of TEVARs performed in the United States increased by 600% from 1998 to 2007, while the total number of thoracic aortic repairs increased by 60%. As larger multicenter trials and meta-analysis studies in the 2000s demonstrate the significant decrease in perioperative morbidity and mortality of TEVAR over open repair, TEVAR became incorporated into standard guidelines. The 2010 American consensus guidelines recommend TEVAR to be “strongly considered” when feasible for patients with degenerative or traumatic aneurysms of the descending thoracic aorta exceeding 5.5 cm, saccular aneurysms, or postoperative pseudoaneurysms. Nowadays, TEVAR is the predominant treatment for degenerative and traumatic descending thoracic aortic aneurysm repair. Although TEVAR has been shown to have decreased early morbidity and mortality compared with open surgical repair, endovascular manipulation of a diseased aorta with endovascular devices continues to have significant risks. Despite continued advancement in endovascular technique and devices since the first prospective trial examined the complications associated with TEVAR, common complications, two decades later, still include stroke, spinal cord ischemia, device failure, unintentional great vessel coverage, access site complications, and renal injury. In this article, we review common TEVAR complications with some corresponding radiographic imaging and their management.

## Introduction

Thoracic endovascular aneurysm repair (TEVAR) was first developed in the early 1990s, and the use of endovascular grafts in thoracic aortic aneurysm repair has since grown to become the mainstay treatment. 1 2 3 Prior to TEVAR, thoracic aortic repairs were done via open surgery for nearly four decades, which conferred 12% mortality rate in elective procedures and more than 50% in emergent open thoracic aortic aneurysm repair. 1 4 Similar high-mortality and -morbidity rates applied to open abdominal aortic aneurysm repairs prior to the advent of endovascular therapy. 5

## History

In search for an alternative treatment for patients with significant comorbidities, Parodi et al 6 first described the feasibility of abdominal aortic exclusion with an endovascular, stented, Dacron prosthetic graft using retrograde access through the common femoral artery for five patients in 1991. In 1994, Dake et al 1 described the first use of a transluminal endovascular graft on the descending thoracic aortic aneurysm in 13 patients. The first TEVAR devices consisted of a customized self-deployable steel Z-stent with the woven Dacron. Each stent was based on each patient's computed tomographic (CT) scan. 1 Further publications on the effectiveness and safety of TEVAR propelled the creation of commercially available devices, which performed well in clinical trials in the late 1990s. 5

**Fig. 1:**
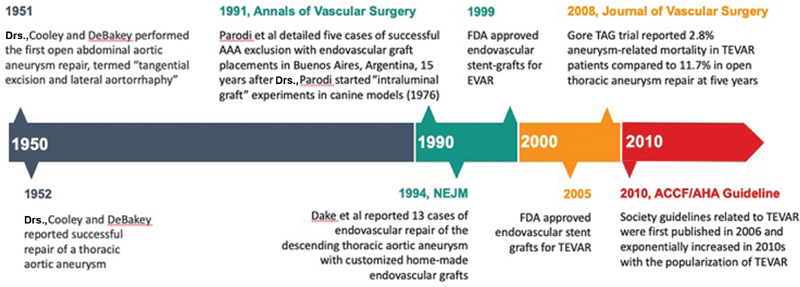
Chronological timeline of the history of thoracic aneurysm repair. FDA, Food and Drug Administration; TEVAR, thoracic endovascular aneurysm repair.

In September 1999, the U.S. Food and Drug Administration (FDA) approved five endovascular grafts for clinical use in abdominal aortic aneurysm repair, but it was not until 2005 that the FDA approved of the use of stent grafts for TEVAR. 5 7 Off-label use of stent grafts on descending thoracic aortic aneurysm slowly grew since 1999. The publication of the Gore TAG trial along with establishing the TEVAR current procedural terminology (CPT) code in 2005 launched TEVAR into mainstream practice ( Fig. 1 ). 4 8 9 10 11 While there were no TEVAR performed in 1998, 9 years later TEVAR rose to 31% of all descending thoracic aortic aneurysm repairs when open repairs dropped to 69% in 2007. 4 Innovations in endograft engineering design, the popularization of endovascular techniques, and improvement in imaging systems have transformed TEVAR into the first-line treatment for thoracic aortic repair in patients with suitable anatomy. 1 It is therefore crucial for clinicians to be familiar to the indications, procedure, and devices, a wide range of complications, and the management of potential complications. 12

## Indications for Thoracic Endovascular Aneurysm Repair

When the FDA approved the use of endovascular grafts for thoracic aortic diseases in 2005, it was limited to repairs of descending thoracic aneurysms, intramural hematoma, and penetrating atherosclerotic ulcer. 9 12 As TEVAR trials and outcomes studies demonstrated the safety of the procedure and decreased mortality and morbidity compared with open thoracic aortic repair, more patients became candidates for the procedure. 2 6 In 2012, FDA expanded graft use on traumatic aortic transection, and then in 2013 for all lesions of the descending thoracic aorta, including Type B dissections. 12

## Guidelines

The 2010 American consensus guidelines recommend that endovascular stent grafting should be “strongly considered when feasible for patients with degenerative or traumatic aneurysms of the descending thoracic aorta exceeding 5.5 cm, saccular aneurysms, or postoperative pseudoaneurysms.” 13 The European guidelines in 2012 recommends TEVAR “when the maximum diameter of the aneurysm exceeds 5.5 cm or if rapid expansion (>5 mm in 6 months) occurs in patients with symptomatic thoracic aortic aneurysm,” taking into account the “patients with increased operative risk” may be considered for a larger aortic diameter threshold. 14

## Preoperative Evaluations

Despite significant advancement in endovascular techniques and device designs, there are certain TEVAR limitations due to patient anatomy and comorbidities. 12 While patients with advanced age and multiple comorbidities should undergo standard preoperative clearance, patients who have significant anatomic challenges require further considerations to prevent complications.

## Anatomic Considerations

Imaging assessment is crucial in preoperative planning. After obtaining computed tomography angiography (CTA) with ≤1 mm cuts from the supra-aortic vessels to the common femoral arteries, the use of reconstructive software for three-dimensional (3D) image rendering is recommended. 10 This allows for detailed assessment of the landing zones, tortuosity, angulation, coverage length, diameter of the aneurysm, involvement of the left subclavian artery, intraluminal thrombus, wall calcification, and access site. 10

A landing zone typically requires a minimum of 20 mm of healthy aortic wall, proximally and distally. 9 10 12 The landing zone inner wall diameter is ideally between 16 and 42 mm, based on the current available device dimensions, and it is recommended to use 10 to 20% oversized grafts to ensure a complete seal and to prevent retrograde aortic dissection. 10 Insufficient proximal landing zone remains challenging for short proximal neck or significant angulation of the aortic arch near the take-off of the left subclavian artery (LSA). 10 12 Techniques, such as hybrid repair with debranching, chimneys, fenestrations and branches, or scallop can be considered. 10 Recently, 3D printed aortic grafts based on preoperative CTA allows for physician-modified fenestrated graft designed for each patient's anatomy, especially in those with poor or insufficient landing zones. 14 Customization of graft design in the future may eventually reduce the difficulty in managing insufficient landing zones.

Access site navigation remains a challenge especially in patients with significant peripheral vascular disease or with tortuous iliac vessels. 10 12 Techniques such as balloon angioplasty can treat stenosis of the iliac arteries. Brachiofemoral through-and-through guidewire techniques can assist graft advancement in tortuous arteries. Retroperitoneal access or iliac conduits can be used to bypass small or occlusive arteries. 10 Other patient factors that should be considered include prior vascular surgeries in the iliofemoral region, but the only major contraindications to TEVAR are current infections at the surgical sites or allergies to the material used in endovascular grafts, for example, patients with known metal allergy might need confirmatory allergy testing to specific metals or need endografts free of the patients' known allergen, commonly nickel. 14 15

## Outcomes and Complications

TEVAR has been shown to have early decrease in perioperative mortality and morbidities; however, studies have not demonstrated evidence for superior long-term outcome over open thoracic aneurysm repair. 9 10 12

A multicenter prospective trial using the GORE TAG Thoracic Endograft in 140 patients from 1999 to 2001 showed a significantly lower rate of spinal cord ischemia, respiratory failure, renal insufficiency, shorter hospital stay, and shorter intensive care unit (ICU) stay in the TEVAR group compared with the open repair group. 8 Although there were three reinterventions in the TEVAR group compared with none in the open group, there was no difference in the overall mortality at 2 years. 2

A meta-analysis review published in 2010 concluded that TEVAR reduced early mortality within 30 days, paraplegia, cardiac complications, transfusions, bleeding that required reoperation, renal dysfunction, pneumonia, and length of stay (LOS). 16 However, there was no significant difference in stroke, myocardial infarction (MI), aortic reintervention, or mortality beyond 1 year. 16

Several studies report favorable long-term outcomes for TEVAR. A single institution 11-year outcomes study in 579 patients between 2004 and 2015 reports that overall survival and aorta-specific survival at 11 years were 45.7 and 96.2%, respectively. 17 Of the 14 patients (7.3%) who required endovascular reintervention, 10 patients had Type I endoleak, two had Type II endoleak, and two had Type III endoleak. 17 There was no report of device failure. Unfortunately, as of yet, there have not been any recent meta-analysis on the long-term device durability for TEVAR.

Despite advancement in techniques and devices in the last two decades, the common complications of TEVAR have not changed significantly ( Table 1 ). 12 14 They include spinal cord ischemia, stroke, endoleaks, access site complications, guidewire injuries, retrograde dissections, renal injury, unintentional great vessel coverage, aortoesophageal and aortobronchial fistulas, and device failure. 9 12 14

**Table 1:** List of TEVAR complications discussed in this article

Complications of TEVAR
Spinal cord ischemia
Stroke
Endoleaks
Endograft collapse
Vascular access and device delivery injuries
Renal failure

Abbreviation: TEVAR, thoracic endovascular aneurysm repair.

## Spinal Cord Ischemia

### Pathophysiology

Paraplegia has been a dreaded complication since the early age of TEVAR, and its rate has not declined despite advancement in techniques and devices. 3 9 10 14 Although TEVAR has proven to be superior to the open surgical repair in many scenarios, it shares a similar rate of spinal cord ischemia (SCI) between 2 and 10%. 3 9 10 14 18 19 20 Spinal circulation has become better understood over the years; the widely accepted pathophysiology behind SCI is from cellular damage from inadequate collateral blood supply to the spinal cord from decrease in blood flow or possible atheroembolism of aortic plaques via segmental arteries that supply the spinal cord. 10 20 In addition to the artery of Adamkiewicz, there is a collateral network of blood supply from adjacent lumbar muscles and the anterior spinal cord artery, including the lumbar, intercostal, subclavian, vertebral, and hypogastric arteries. 10 21 Although much controversy exists regarding the optimal preventative methods and treatment for SCI, the main strategies aim to increase the mean arterial pressure (MAP) and placing a lumbar drain to drain cerebrospinal fluid (CSF) to optimize perfusion of the spinal cord. 20 22 23

### Risk Factors

**Fig. 2:**
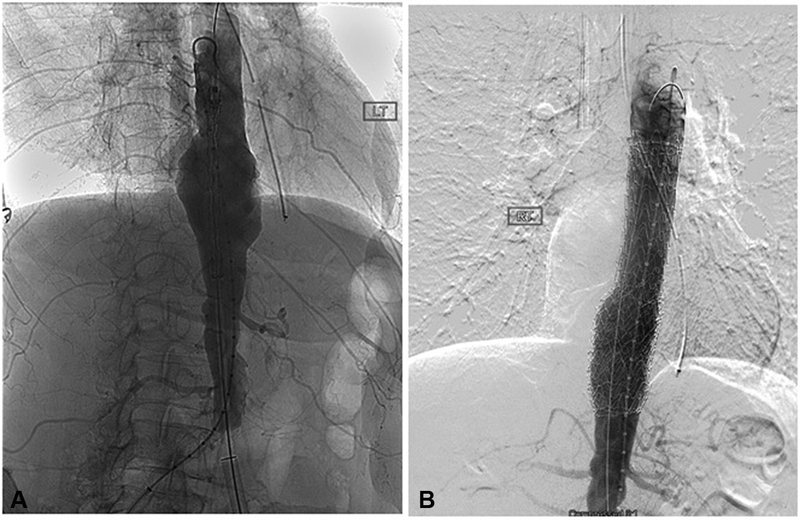
( **A** ) Conventional angiography of the aorta shows an aneurysm at the level of the diaphragm. ( **B** ) Note the occlusion of radicular arteries upon deployment of the endograft on this digital subtraction image.

Risk factors for SCI can be classified by patient-related or surgery-related factors. 20 The patient-related factors, including degenerative aneurysms, advanced age, chronic obstructive pulmonary disease (COPD), hypertension (HTN), and renal failure, contribute to the extent of aneurysm severity or increased chance of perioperative hypotension. 24 25 26 27 28 The surgery-related risk factors involve any intervention that would decrease spinal collateral flow, especially in the lumbar region where there is less robust collateralization. These include excessive blood loss, coverage of LSA or hypogastric artery, coverage of greater than two arteries that supply the collateral network, longer procedure duration, and total aortic coverage greater than 200 mm ( Fig. 2 ). 26 28 29 30

### Prevention and Management

There are numerous proposed neuroprotective strategies in the literature. 10 General concepts include enhancing spinal cord perfusion, pharmacologic neuroprotection, and reducing metabolic and oxygen requirements. 10

There are many strategies to enhance spinal cord perfusion. First, it is recommended to maintain an elevated intraoperative and postoperative MAP between 85 and 100 mm Hg. 10 31 For patients who suffer SCI, MAP greater than 90 mm Hg is preferred. 29 Second, clinicians should be mindful of the extent of graft coverage to preserve as much collateral blood supplies as possible. 10 31 Third, pre-TEVAR LSA revascularization has been shown to decrease the risk of SCI if LSA is likely to be covered by graft. 31 Fourth, CSF drainage improves spinal cord perfusion by decreasing intrathecal pressure and increasing the pressure gradient. 23 31 32 33 Although a meta-analysis showed no benefit in prophylactic CSF drainage in TEVAR patients, 33 some studies show evidence for prophylactic CSF drainage in patients with prior abdominal aortic aneurysm (AAA) repair, extensive thoracic aortic coverage, and subclavian artery coverage without revascularization. 31 32 33 34 35 Lastly, clinicians are investigating novel procedural strategies to induce remodeling of the spinal collateral blood flow with controlled partial ischemia. 10 Studies have shown the benefit of performing a staged repair in patients undergoing extensive TEVAR, especially in hybrid thoracoabdominal aortic aneurysm repairs, to stimulate collateral arterial supply remodeling after initial partial coverage and then complete the full graft coverage at a later time. 10 36 This may be considered when covering a large segment greater than 30 cm. 30 Other studies have used minimally invasive segmental artery coil embolization (MISACE) to stimulate arteriogenesis around the embolized segmental arteries that perfuse the spinal cord prior to the TEVAR procedure. This is thought to precondition the spinal cord for ischemia during the actual repair. 28 An ongoing clinical trial—Paraplegia Prevention in Aortic Aneurysm Repair by Thoracoabdominal Staging with “Minimally-Invasive Segmental Artery Coil-Embolization”: A Randomized Controlled Multicenter Trial (PAPA-ARTIS)” that involves multiple medical centers in Europe and United States—is evaluating the effectiveness of MISACE. The temporary aneurysm sac perfusion procedure that prevents immediate aneurysm sac thrombosis is another potential solution to the problem of SCI in TEVAR. It was proven successful in a small clinical trial, but more studies are required to determine the feasibility. 37

Other medical adjunct measurements have also been popular in practice. 10 19 Pharmacologic agents, such as intrathecal papaverine injection that induces vasodilation around the spinal cord circulation, have been shown to reduce the rate of paraplegia in a prospective randomized study. 38 A study of combined neuroprotective protocol of perioperative naloxone, intraoperative mannitol, and steroids along with mild hypothermia reports a slightly reduced rate of SCI (0.65%; 1 out of 154 patients). 19 In theory, naloxone reduces the release of neurotransmitter, mannitol reduces spinal cord swelling, steroids have a stabilization effect on neural cell membranes, and mild hypothermia lowers the metabolic and oxygen requirement; altogether increasing the body ischemic tolerance. 19 It is unclear if each of the elements has a significant effect in preventing SCI.

## Stroke

Stroke continues to be a major complication of TEVAR, with reported incidences of stroke after TEVAR ranging from 1.2 to 8.2%. 10 14 26 39 TEVAR eliminates the embolism risk from aortic cross-clamping or cardiopulmonary bypass, but TEVAR can cause embolization from the manipulation of a diseased aortic arch and great vessels with wires and catheters. 10 14 Risk factors for embolic stroke include acute aortic dissections, large atherosclerotic burden of the aortic arch, HTN, and known cerebrovascular disease. 10 40 Besides embolic events, reduction in global cerebral perfusion is another main cause of stroke perioperatively. 14 The risk increases with increased aortic coverage, occlusion or coverage of the LSA, perioperative hypotension, and prolonged surgery. 10 12 15 40

To prevent perioperative stroke, some studies propose screening patients for a dominant left vertebral artery with brain CTA or magnetic resonance imaging (MRI), especially in patients with significant comorbidities. 10 12 41 Feezor et al 39 reported a decreased stroke rate after TEVAR with preoperative LSA revascularization. On the other hand, a meta-analysis showed no significant difference in neurologic complications or mortality with preoperative LSA revascularization. 41 New devices, such as fenestrated grafts, appear to be promising solutions to prevent occluding branches of the aortic arch, but more studies are needed to support the benefit of these devices. 42 43

**Fig. 3:**
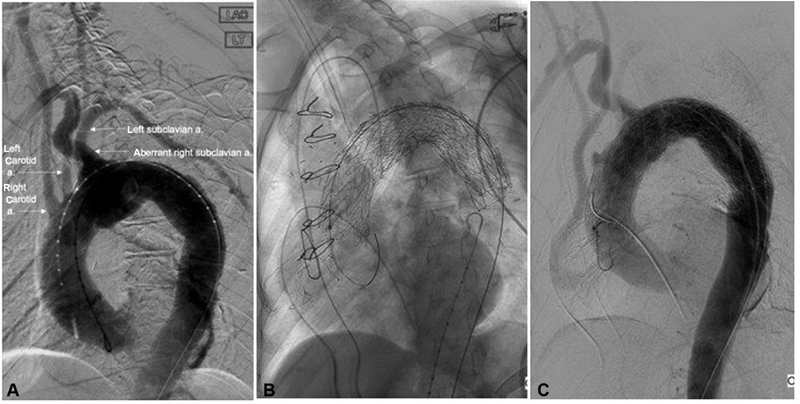
Endograft covering ( **A** ) aortogram prior to deploying thoracic aortic stent shows an aberrant right subclavian artery originating distal to the left subclavian artery, along with the right and left carotid arteries (labeled with arrows). Note that, this is different from the most common aortic arch anatomy with three great vessels originating from the aortic arch: brachiocephalic, left common carotid, and left subclavian arteries. ( **B** ) The thoracic aortic stent covering the origin of all three vessels after placement. ( **C** ) Aortogram after pre-TEVAR extra-anatomic bypass from the root of the aorta to the left carotid artery. a., artery; TEVAR, thoracic endovascular aneurysm repair.

When proximal endograft coverage is needed and causes occlusion to the great vessels branching off of the aorta ( Fig. 3B ), pre-TEVAR revascularization, in this case, a series of extra-anatomical bypasses, is required to maintain cerebral blood flow. Fig. 3C shows perfusion of the carotid artery through the extra-anatomic bypass.

## Endoleaks

Endoleak is defined by the persistence of blood flow and pressurization of the diseased aortic segment that is supposed to be excluded by the endograft. 43 Since the term “endoleak” was first published in 1997, the incidence of endoleak decreased from 20–50% to 5–10% with modern devices. 12 44 45 46 47 48 Endoleaks are classified according to the mechanism of persistent pressurization of aneurysmal sac. Each type of endoleak corresponds with a different management strategy ( Table 2 ). 48 Endovascular intervention remains the mainstay of therapy for endoleak repair. Treatments aim to either bridge the endoleak defect or to embolize the endoleak source such as a lumbar artery. 48 Open surgical repair sometimes is necessary when endovascular techniques fail.

**Table 2:** Summary of different types of endoleaks with respective definitions and treatment modalities when repair is indicated

Type	Mechanism	Therapy
I	Sealing failure at one of the attachment sites of the graft to the vessel. Ia: proximal leak; Ib: distal leak	Endovascular repair
II	Retrograde flow through collateral vessels into the perigraft space	Observation vs. endovascular repair
III	Device failure due to dysfunction of the components of a modular graft (IIIa) or in the fabric of the graft (IIIb)	Endovascular repair (very rare)
IV	Passage of blood or fluid into the aneurysmal sac as a result of graft porosity	Endovascular repair (very rare)
V	Continued aneurysm sac expansion without a demonstrable leak by an imaging modality	Observation vs. endovascular repair

Type I and III endoleaks are relatively high-pressure systems due to continued communication between systemic circulation and the aneurysm. 48 49 Therefore, Type I and III endoleaks have a greater risk of rupture and require intervention. 48 Type I endoleaks can occur immediately after graft placement or develop over time, and they are commonly detected via contrast-enhanced CT on postoperative follow-up imaging. 12 48 Delayed onset of Type I endoleak can be associated with short or angulated proximal aortic neck anatomy that prevents a proper seal. 50 Endovascular therapies aim to create an effective seal between the stent graft and the aorta. 51 Novel techniques such as EndoAnchors used in the ANCHOR study demonstrated effectiveness as a prophylaxis to prevent Type Ia endoleak; however, EndoAnchors as the sole endovascular treatment for Type Ia endoleak leaves 34% of patients with persistent endoleak. 51 If mechanical approaches fail, there are several commercially available substances to embolize the endoleak. 48 Type III endoleak is usually caused by insufficient overlapping between graft components, which is treated with additional devices to seal the defect. 48

Type II endoleak is the most common type, accounting for approximately 75% of all endoleaks in the EVAR literature, and its treatment is variable. 47 Most of the Type II endoleaks will remain stable, decrease in size, or spontaneously thrombose over time. 45 Treatment is required if symptoms persist or if an aneurysm expands, frequently with endovascular embolization of the supplying blood vessel. 10 12 52

With the advancement in graft material, Type III and IV endoleaks have become very uncommon and are usually treated with additional stent grafts if needed. 52 Type V endoleaks are more elusive. Some authors have reported successful outcomes converting polytetrafluoroethylene grafts to polyester grafts, implanting proximal and distal extension cuffs, aspiration, and laparoscopic fenestration of the aneurysm sac. 53 54 Most cases of Type V endoleaks with expansion of the aortic aneurysm require open repairs.

## Endograft Collapse

Stent-graft collapse after TEVAR is a rare complication that has been reported in a few cases with predominantly young patients who were treated for traumatic aortic dissection. 10 12 54 Muhs et al 55 concluded that smaller distal aortic diameter and minimal intragraft aortic diameter are risk factors for endograft collapse. Kasirajan et al 56 reviewed all cases of TAG device collapse (compression or infolding) from 1998 to 2008 and concluded that most of the cases were caused by off-label use in trauma patients and endograft oversizing, where endografts with diameters 15 to 30% larger than the intraluminal diameter are placed. The incidence of stent collapse is rare but with serious morbidity and mortality that require emergent TEVAR or open repair. 12 57 58

## Vascular Access and Device Delivery Injuries

Although TEVAR has revolutionized the treatment of aortic disease, vascular access and device delivery are still the limiting factors for some patients. 10 Devices can be delivered in a retrograde or anterograde fashion. Both approaches have several options and techniques. 59 60 61

**Fig. 4:**
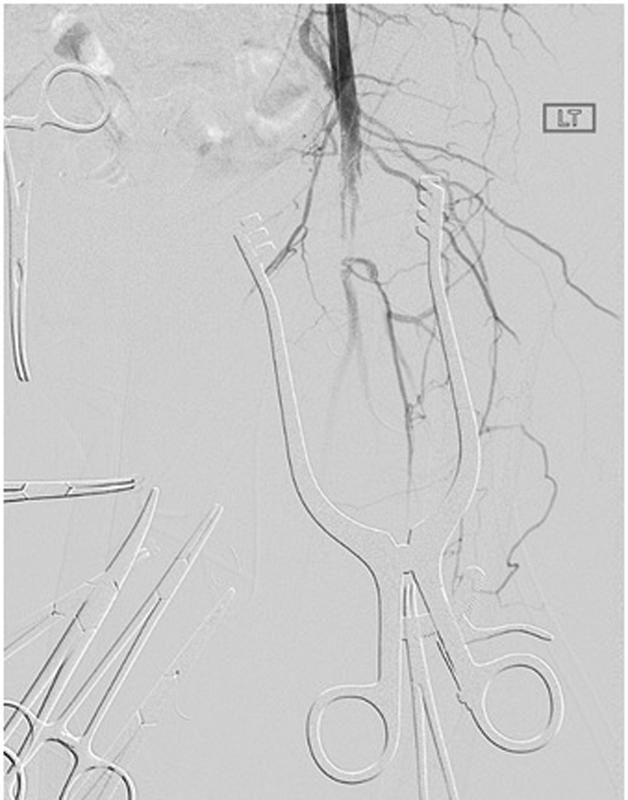
Dissection of the iliac artery at the access site.

**Fig. 5:**
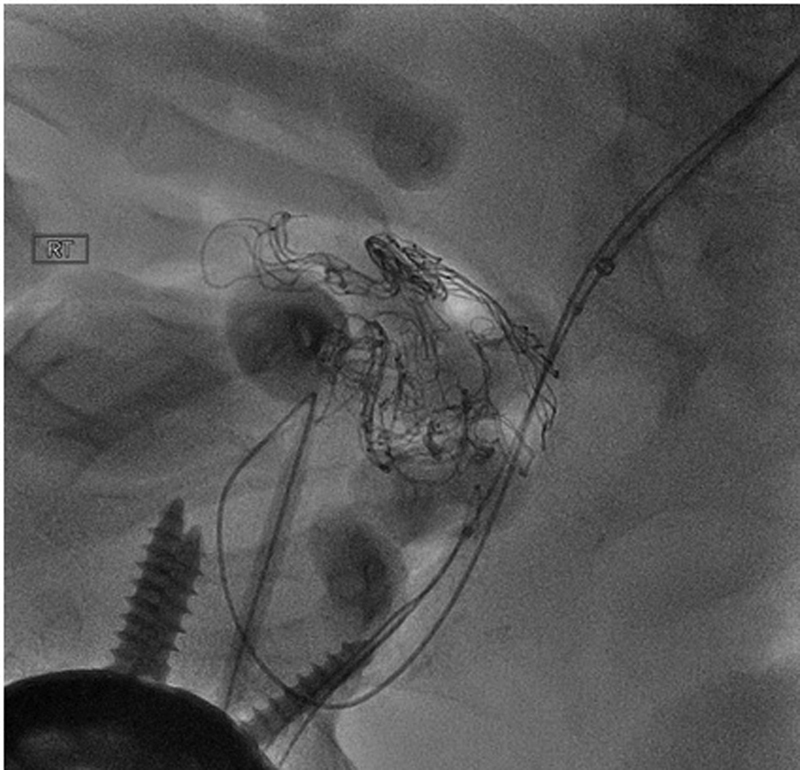
Example of guidewire injury. Guidewire is shown to have perforated the vessels, demonstrated with contrast extravasation.

**Fig. 6:**
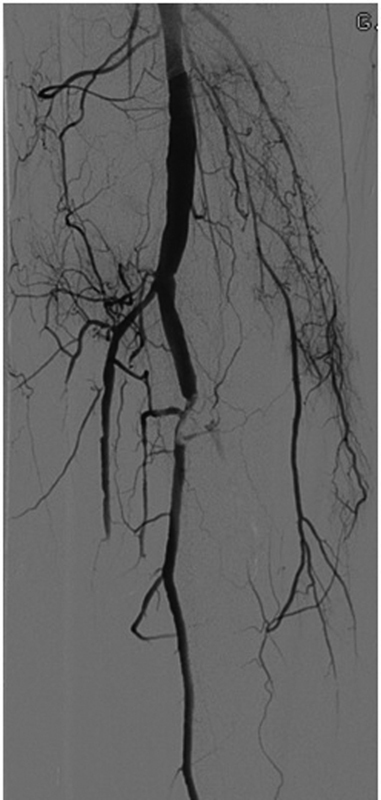
Distal embolization to tibioperoneal trunk following a thoracic endovascular aneurysm repair.

**Fig. 7:**
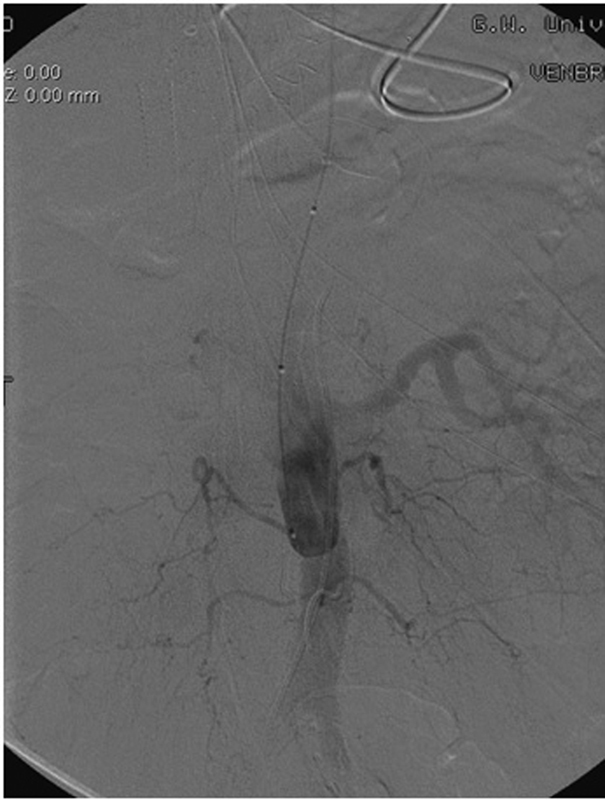
Mesenteric and renal ischemia from aortic dissection. Aortogram with flush catheter in compressed true lumen supplying the left renal.

TEVAR devices are most commonly delivered in a retrograde manner via the iliofemoral vessels. 10 12 The iliofemoral access is dependent on sheath size, average iliac artery diameter, iliofemoral morphology, and preoperative ankle-brachial index. 62 Early complications include arterial dissection ( Fig. 4 ), iliac artery rupture, arterial perforation ( Fig. 5 ), and distal thromboemboli ( Fig. 6 ). 39 Arterial disruption can cause severe retroperitoneal hemorrhage and require rapid conversion to open repair, and retrograde arterial dissection can cause mesenteric or renal ischemia ( Fig. 7 ) that require emergent endovascular or open repair. The most common late complication was lower limb ischemia that needs immediate medical treatment and potentially surgical bypass. 39

For patients with vascular occlusive diseases or other factors that prohibit the retrograde delivery of the stent graft devices, an antegrade approach may be feasible. 12 Roselli et al 60 described three different techniques for the anterograde delivery of devices: axillary artery, ascending aorta, or direct placement. The main complications were stroke, spinal cord injury, respiratory failure, and renal failure. 61

## Renal Failure

Although TEVAR avoids aortic cross-clamp or cardiopulmonary bypass, acute kidney injury (AKI) is still a common complication after TEVAR, ranging between 1 and 34%. 12 The wide range of variability is due to the lack of standardized definition for renal failure. 63 Besides patient factors, such as hypertension and chronic renal failure that increases the risk of AKI, iodized contrast is widely accepted as a contributing factor of AKI. 64 Preventive strategies, such as preoperative hydration and administering N-acetylcysteine, have been widely used but with varying results. 65

## Conversion to Open Repair

The incidence of patients who require conversion to open thoracic aortic aneurysm repair from TEVAR has been reported to be 2.2 to 7.2% at experienced centers. 66 67 In a retrospective study by Canaud et al, 68 14 patients out of 236 required open surgical repair after TEVAR for retrograde Type A dissection, secondary aortobronchial fistula, stent-graft infection, aortoesophageal fistula, aneurysm enlargement, and stent-graft collapse. With the advent of TEVAR, it is even more important for clinicians to recognize situations that require rapid conversion to open repair.

## Conclusion

TEVAR has transformed the prognosis for patients with thoracic aortic aneurysm and become the gold standard for elective thoracic aortic aneurysm repairs. While this article belabors the importance of understanding the wide range of TEVAR complications and limitations, it is also important to glance back in the history of aortic aneurysm repair and appreciate how far endovascular and open surgical techniques have advanced. With long-term follow-up data from TEVAR in the next decade, clinicians and engineers will be able to continue to refine endovascular technology and redefine the applications for endovascular techniques.
